# Variations in Plant Richness, Biogeographical Composition, and Life Forms along an Elevational Gradient in a Mediterranean Mountain

**DOI:** 10.3390/plants10102090

**Published:** 2021-10-01

**Authors:** Letizia Di Biase, Loretta Pace, Cristina Mantoni, Simone Fattorini

**Affiliations:** Department of Life, Health and Environmental Sciences, University of L’Aquila, Via Vetoio, 67100 L’Aquila, Italy; letizia.dibiase@graduate.univaq.it (L.D.B.); lorettagiuseppina.pace@univaq.it (L.P.); cristina.mantoni@univaq.it (C.M.)

**Keywords:** altitude, Apennines, biogeography, chorotypes, Italy, latitude, life forms, Raunkiaer, species richness

## Abstract

Despite the increasing interest in elevational patterns in biodiversity, few studies have investigated variations in life forms and biogeographical composition, especially in the Mediterranean biome. We investigated elevational patterns in species richness, biogeographical composition (chorotypes) and life forms (Raunkiaer classification) along an elevational gradient in a Mediterranean mountain (Central Italy). We found a general hump-shaped pattern of species richness, which can be explained by harsher conditions at the lowest and highest elevations. This pattern is distinctly related to prevalence at mid elevations of species with European and Euro-Asiatic distribution, which are favored by a temperate climate. Phanerophytes and geophytes (which are mainly associated with woods) were concentrated at mid elevations where woodlands prevail. Hemicryptophytes increased with elevation, consistently with their ability to cope with high altitude climatic conditions. Mediterranean species declined with elevation because they are negatively affected by decreasing temperatures. Chamaephytes showed a U-shaped pattern, suggesting they are able to cope with arid and cold conditions at the extremes of the gradient. Endemics increased with elevation because of their association with mountainous areas as key places for endemism evolution. These results illustrate how elevational patterns in species richness, biogeographical composition and life forms are interrelated and demonstrate reciprocal insights for understanding current vegetation settings.

## 1. Introduction

The progressive changes in many factors such as temperature, precipitation, and soil characteristics that occur with elevation in mountain areas provide unique chances to investigate how biodiversity varies in response to environmental factors within geographically restricted areas [1,2,3,4,5,6,7,8,9,10,11,12,13,14,15,16,17,18,19,20,21,22]. Since species responses to environmental factors are mediated by their traits and are reflected by their distribution, vegetation studies frequently take advantage by classifying species into life forms [23,24,25,26,27] and biogeographical categories [28,29,30,31,32]. Surprisingly enough, however, only a few studies have investigated variations in plant biogeographical [18,33] and life form (e.g., [18,20,33,34,35,36,37,38,39,40,41,42]) composition along elevational gradients.

A common approach to express the biogeographical composition of a species assemblage relies on the concept of chorotype. Chorotypes refer to groups into which species with similar distributions can be classified [43,44,45]. Chorotypes are established by an inductive and recursive process in which species distributions are mapped, their contours are compared, and species with similar ranges are classified with the same group, i.e., they form a chorotype [43,44,46]. After a chorotype is defined by the overlap of multiple species distributions, any other species showing a similar distribution can be assigned to that chorotype. Species that are classified under the same chorotype can belong to completely unrelated taxa or ecological groups. Thus, chorotypes can be assimilated into Morrone’s concept of biota [47]. As “abstractions” used to express recurrent species distributions, chorotypes are also roughly similar to the “generalized tracks” of Croizat [48], although, in the case of chorotypes, no shared history is implied. Chorotypes are widely used both to shortly indicate species distributions and to make hypotheses about the origin of plant and animal assemblages [43,44,46]. As species with similar distributions should also have similar macroecological needs [31], the analysis of the chorotype composition of local species assemblages can be used to draw inferences about which ecological and historical factors have shaped such assemblages. For instance, the presence of species with Arctic distributions in the Alps, or that of species with Boreo-Alpine distributions in the Apennines, is attributed to Pleistocene glacial colonization [49]. Biogeography of mountain areas may be particularly complex because of their isolation (which promotes speciation and endemism), their unique geological histories and the effects of paleoclimatic events [21,50]. Thus, the study of variation in chorological composition along elevational gradients may give important insights to understand how biogeographical factors have contributed to shape current communities [51].

Life forms are widely used in plant community ecology as they make it possible to compare plant assemblages with different taxonomical composition [24,25,26]. Many functional classifications, based on morphological, phenological and physiological features, have been proposed [52] to group plant species into classes based on similarity of function and structure. However, Raunkiaer’s life forms [53], despite some criticism [54], are the most accepted and used classification system [35,55] because of its inherent simplicity and parsimony. This classification system groups terrestrial plants into the following major groups according to the protection strategies during the unfavorable season and the height of the renewable buds in relation to the soil surface: therophytes (annual plants surviving harsh conditions as seeds), hemicryptophytes (plants with buds at or near to soil surface), chamaephytes (plants with buds within 0.25 m above the soil surface), phanerophytes (plants taller than 0.25–0.5 m, with buds on aerial shoots) and geophytes (plants with belowground buds as storage organs, i.e., rhizomes or bulbs) [56,57]. Since these characteristics are genetically determined and reflect different tolerances towards climatic variables, Raunkiaer’s classification can be used to investigate evolutionary processes that shape species assemblages and drive species’ morphological traits [25,58,59,60]. These features make Raunkiaer’s system a very useful and practical tool for ecological classification of plant communities [27,57].

Raunkiaer’s classification has been introduced for temperate regions, where the position of the regenerative buds has a pivotal role in allowing plant survival during the unfavorable season, but it can be profitably used also in warm climates, where drought and heat represent important limiting factors for plant growth [40,61]. For example, the Mediterranean biome is characterized by a strong seasonality, with stressful conditions in summer due to high temperature and virtually absent rainfall [62]. At high altitudes, however, plants must cope with very cold winter conditions even in the Mediterranean biome. Mediterranean mountains are thus characterized by an emphasized “double stress” gradient, with high temperatures and drought stress limiting plant growth at lower altitudes, especially in summer, whereas low temperatures and winter’s frost stress are important limiting factors at high elevations [21,22,63,64].

Mountains in the Mediterranean environment are therefore excellent natural laboratories to investigate how chorotype and life form composition of plant assemblages change along the elevational gradient. However, very few research studies have investigated variation in chorological [33] and life form [20,33,41] composition along elevational gradient in Mediterranean mountains. Moreover, various sources of complexity influenced these studies, making comparisons and interpretations difficult. For example, both Lazarina et al. [20] and Di Musciano et al. [41] used elevational gradients extended over wide areas. When data are aggregated from sites dispersed over a wide area, the resulting elevational gradient may represent a heterogeneous combination of species assemblages belonging to floras influenced by different biogeographical and evolutionary processes operating on large scales. To avoid these problems, it is important to focus on elevational gradients in geographically narrow areas. However, even in narrow scale approaches, a common procedure is that of using elevational bands and assuming that species are continuously distributed along the gradient, from the lowest to the highest belt [33,41]. This could lead to consider coexisting species that, although recorded at the same elevation, occupy different areas and do not form true communities. Finally, observed patterns can be incomplete if the study involved only a section of the gradient. For example, Theurillat et al. [33] used a gradient that starts from about 1000 m, well beyond the typical mid elevations of Apennine chains (about 500 m).

In this paper, we used data from a small Mediterranean mountain (Mount Genzana, a protected area in the Abruzzi Region, Central Apennines) to investigate how species richness, biogeographical composition and life forms vary along an elevational gradient. As far as we are aware, this is the first study that used plot data instead of data aggregated and interpolated at the belt level (thus avoiding the risk of assuming as co-occurring species that lives at the same elevation but in different places) along a complete elevational gradient (from lowland to mountain summit, thus avoiding the risk that the pattern is biased towards a part of the gradient) within a small geographic region (thus avoiding the risk of mixing different floras).

Specifically, we tested the following predictions:

(1) Many research studies dealing with the species-elevation relationship showed a unimodal pattern with high richness at intermediate altitudes, but monotonic decreases were also frequently reported [4,15,21,65,66,67,68,69,70,71,72,73]. Thus, we tested whether in our study system variation in species richness followed a unimodal or a monotonically decreasing pattern and used information on biogeographical composition and life forms to put forwards possible explanations for the observed pattern.

(2) We made specific predictions about variation in the proportion of the various chorotypes with elevation. Given the inner position of the study area, we hypothesized a continental character of the flora, with a predominance of species more or less widely distributed in the temperate areas of Europe and Asia. Based on their association with temperate climatic conditions [74], we predicted that these species should be especially numerous in the middle of the gradient, thus contributing to generating a hump-shaped pattern in the species richness. As the species with a Mediterranean distribution are those more or less strictly associated with the Mediterranean climate [74], we also predicted that Mediterranean species should be mostly restricted to low elevation areas, rapidly declining with increasing elevation. Because of the pivotal role exerted by mountain areas in promoting endemism through isolation [21], most of endemics in the Italian peninsula are montane species [75,76], and hence we predicted that their proportion should increase with elevation. We also predicted that orophytes in general should increase their proportion with elevation.

(3) Regarding the life forms, we predicted that, because of the mountainous character of the study area, the therophytes, which are a predominant group in low Mediterranean areas with thermo-xeric climatic conditions [6,20,40,41], will be the less represented group. By contrast, we predicted a dominance of hemicryptophytes and chamaephytes, which are the most represented life forms in high mountain ecosystems [6,33,38,40,42,77]. In accordance with their ability to cope with cold climate due to their buds near the soil surface [78], we predicted an increase in the proportion of hemicryptophytes with elevation. We also predicted an increase of chamaephytes with elevation because they are able to overwinter due to the position of their buds, which allows them to benefit from the higher temperature of the soil and the protection of the snow that covers them during winter [6]. However, we expected that chamaephytes represent a relatively high fraction also of lowland plant assemblages because of their tolerance to drought. For the phanerophytes, which are mainly represented by trees, we predicted a hump-shaped pattern, because in the study area woodlands are concentrated at intermediate elevations in response to climatic factors [79]. Finally, as in Italy the geophytes are mainly associated with deciduous mixed woodlands and beech forests [74], we predicted for this group an analogous hump-shaped pattern.

(4) Since climate patterns are similar along elevational and latitudinal gradients, typical lowland vegetation formations are similar to those of lower latitudes, whereas typical highland vegetation is similar to that of higher latitudes [3,21]. Thus, one could ask if patterns of variation in life forms observed along the elevational gradient are similar to those that occur along a latitudinal gradient. A parallelism between elevational and latitudinal variations in Raunkiaer’s life form composition has been known for decades (see, for example, [80,81,82]), yet formal studies still seem to be lacking. Thus, to test the hypothesis of this parallelism, we modelled patterns of latitudinal variation in life form composition along the Italian peninsula and compared them with the observed elevational patterns.

## 2. Results

**Figure 1:**
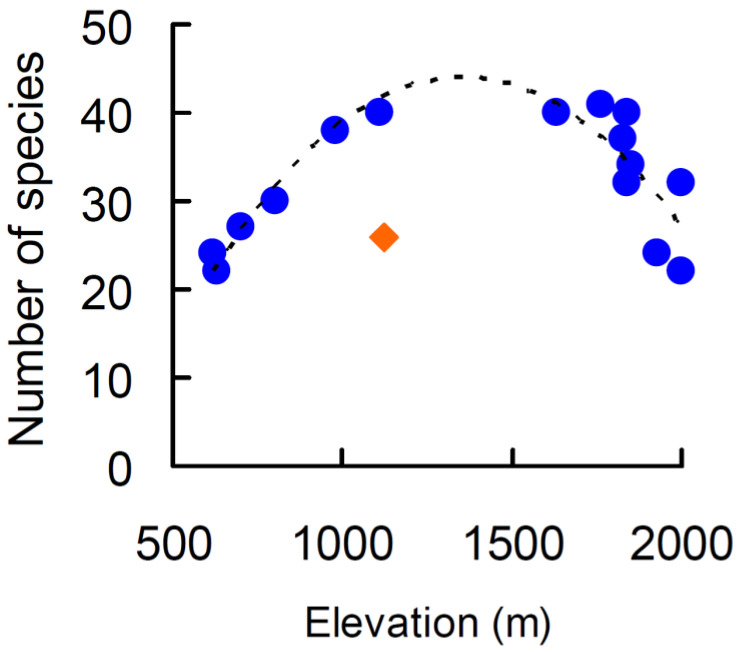
Relationship between plant species richness and elevation on a Central Apennine mountain (Mount Genzana) fitted with a parabolic model. Diamond indicates an outlier excluded from the fit.

Species richness followed a hump-shaped pattern (Figure 1; *y* = −3.011 × 10^−5^*x*^2^ + 0.083*x* − 17.722, *R*^2^ = 0.526, *P* = 0.008). When the *c*-values were used, the relationship became non-significant (*y* = −1.387 × 10^−5^*x*^2^ + 0.0394*x* + 9.775, *R*^2^ = 0.217, *p* = 0.203). However, the low goodness-of-fit values were due to an outlier represented by a relevé with a very small number of species (26 species at 1120 m). If this point is omitted from regressions, both the parabolic model using species richness (*y* = −4.096 × 10^−5^*x*^2^ + 0.111*x* – 31.079, *R*^2^ = 0.772, *p* < 0.0001) and that based on *c*-values (*y* = −2.460 × 10^−5^*x*^2^ + 0.067*x* − 3.432, *R*^2^ = 0.523, *p* = 0.012) increased their goodness-of-fit and were significant.

The chorological spectrum was dominated by the European species (22.4% of sampled species), followed by the Euromontane (18.0%), Endemic (13.6%), Eurymediterranean (12.4%) and Euro-Asiatic (10.4%) ones. Other chorotypes had percentages lower than 10%. Namely, the Cosmopolitan species accounted for 5.6% of the sampled flora, the Mediterraneo-Montane species for 5.2%, and the Boreal species for 4.4%; both the Stenomediterranean and the Paleotemperate species were 4% of the sampled flora.

**Figure 2:**
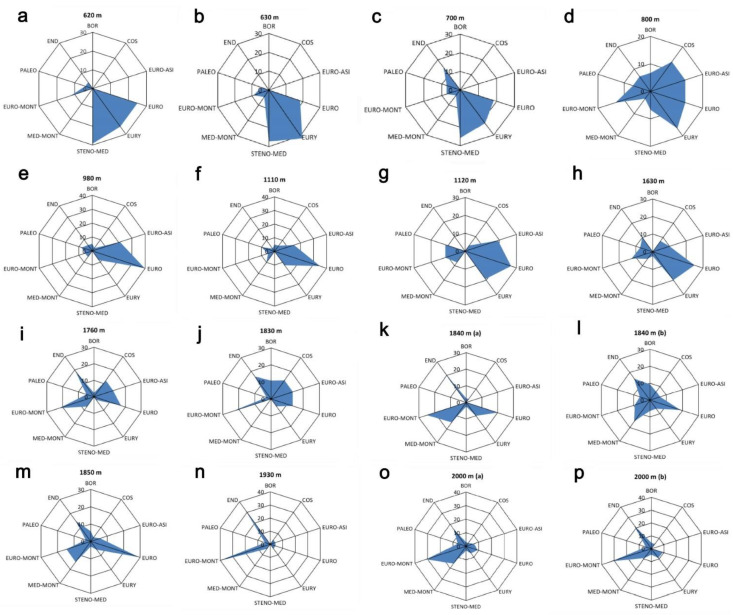
Percentage of different chorotypes found in plant assemblages along an elevational gradient on a Central Apennine mountain (Mount Genzana): (**a**) 620 m, (**b**) 630 m, (**c**) 700 m, (**d**) 800 m, (**e**) 980 m, (**f**) 1110 m, (**g**) 1120 m, (**h**) 1630 m, (**i**) 1760 m, (**j**) 1830 m, (**k**) 1840 m, (**l**) 1840 m, (**m**) 1850 m, (**n**) 1930 m, (**o**) 2000 m, (**p**) 2000 m. Chorotypes: BOR = Boreal, COS = Cosmopolitan, EURO-ASI = Euro-Asiatic; EURO = European; EURY = Eurymediterranean; STENO-MED = Stenomediterranean; MED-MONT = Mediterraneo-Montane; EURO-MONT = Euromontane; PALEO = Paleotemperate; END = Endemic. For 1840 m and 2000 m elevations, two relevés (conventionally indicated, in both cases, as (**a**) and (**b**)) were available.

The relative contribution of the different chorotypes varied distinctively with elevation (Figure 2). At lower elevations (below 800 m), plant assemblages were dominated by Stenomediterranean and Eurymediterranean species (Figure 2a–c). At around 800 m, we observed a strong reduction in the Stenomediterranean species and the appearance of the Euro-Asiatic species (Figure 2d). Assemblages from around 1000 m to 1700 m were dominated by Euro-Asiatic and European species (Figure 2e–h). Over 1700 m, assemblages are mainly characterized by large proportions of Endemic species (Figure 2i–p).

**Figure 3:**
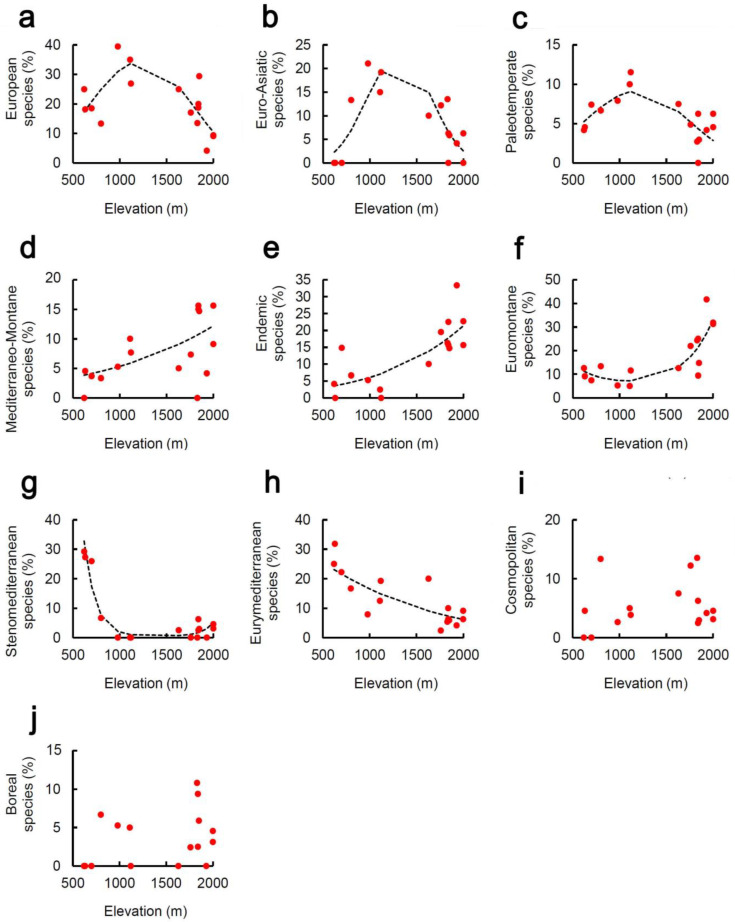
Relationship between percentage of chorotypes and elevation on a Central Apennine mountain (Mount Genzana): (**a**) European species, (**b**) Euro-Asiatic species, (**c**) Paleotemperate species, (**d**) Mediterraneo-Montane species, (**e**) Endemic species, (**f**) Euromontane species, (**g**) Stenomediterranean species, (**h**) Eurymediterranean species, (**i**) Cosmopolitan species, (**j**) Boreal species. Dashed lines are parabolic or monotonic fit obtained with generalized linear models. Fit parameters are reported in Table 1.

Proportion of European (Figure 3a, Table 1) and Euro-Asiatic (Figure 3b, Table 1) species followed a hump-shaped pattern, thus increasing at intermediate elevations. This pattern is also characteristic of Paleotemperate species (Figure 3c, Table 1). Proportion of Mediterraneo-Montane (Figure 3d) and the Endemic (Figure 3e, Table 1) species increased monotonically with elevation, whereas the Euromontane species (Figure 3f, Table 1) varied with elevation with a parabolic pattern, which indicates that these species are concentrated at the lowest and, more markedly, at the highest elevations. Proportion of Stenomediterranean species decreased rapidly from 600 to 800 m (Figure 3g, Table 1). Proportion of Eurymediterranean species decreased monotonically with elevation (Figure 3h, Table 1). Proportion of Cosmopolitan (Figure 3i) and Boreal (Figure 3j) species did not show any pattern.

**Figure 4:**
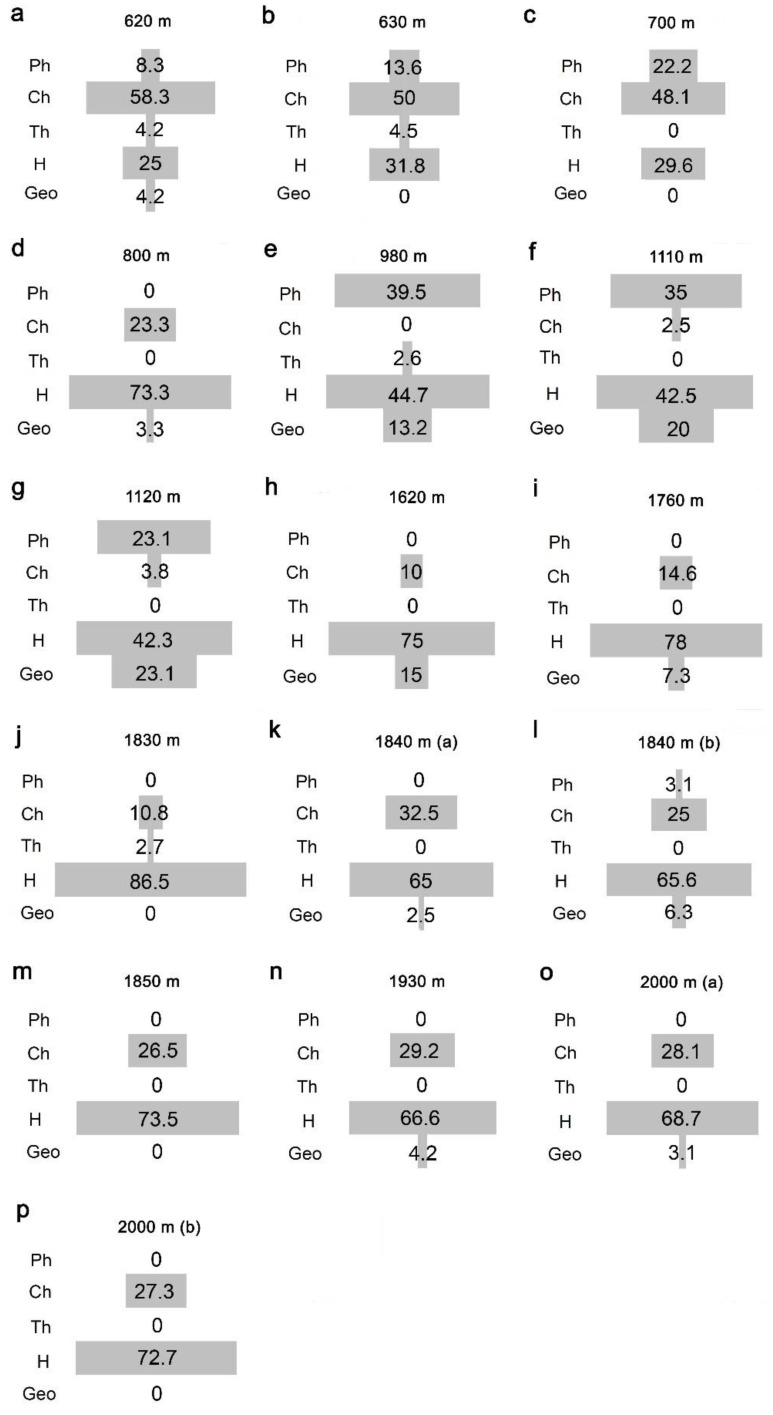
Life form composition of plant assemblages along an elevational gradient on a Central Apennine mountain (Mount Genzana): (**a**) 620 m, (**b**) 630 m, (**c**) 700 m, (**d**) 800 m, (**e**) 980 m, (**f**) 1110 m, (**g**) 1120 m, (**h**) 1630 m, (**i**) 1760 m, (**j**) 1830 m, (**k**) 1840 m, (**l**) 1840 m, (**m**) 1850 m, (**n**) 1930 m, (**o**) 2000 m, (**p**) 2000 m. Life forms: Ph = Phanerophytes, Ch = Chamaephytes, Th = Therophytes, H = Hemicryptophytes, Geo = Geophytes. Numbers indicate percentages. For 1840 m and 2000 m elevations, two relevés (conventionally indicated, in both cases, as (**a**) and (**b**)) were available.

Species frequencies varied significantly among the life forms (chi-square test for deviation from uniform distribution: χ^2^ = 254.800, *df* = 4, *p* < 0.0001): the hemicryptophytes were the prevailing group, with 58.8% of the sampled species; chamaephytes represented 18.4% and phanerophytes 12.4% of the sampled flora, respectively. Geophytes represented 9.2% of the sampled species, and therophytes only 1.2%. Proportions of life forms in the study area were significantly different from those recorded from Italy (χ^2^ = 100.229, *df* = 4, *p* < 0.0001; the Italian spectrum is as follows: hemicryptophytes: 41.7%; therophytes: 25.1%; geophytes: 12.1%; chamaephytes: 10.3%; phanerophytes 8.5%.; hydrophytes: 2.3%; helophytes: 0.3%). The relative contribution of the different life forms to species assemblages varied distinctively with elevation (Figure 4). Chamaephytes were mostly abundant at the lowest (<800 m, Figure 4a–c) and highest (>1800 m, Figure 4k–o) elevations. Hemicryptophytes were particularly abundant from 1600 m (Figure 4h–p). Phanerophytes were mostly concentrated at around 1000 m (Figure 4d–g) and a similar pattern occurred for the geophytes (Figure 4e–g). Therophytes were rare everywhere, and mostly limited to low elevations (Figure 4a,b).

**Figure 5:**
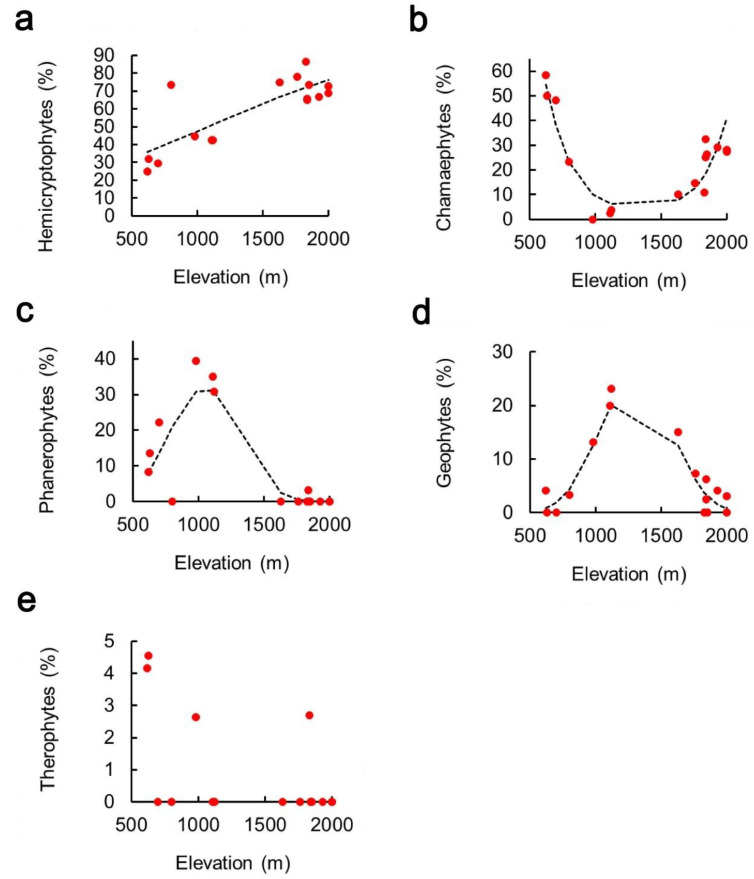
Relationship between percentage of life forms and elevation on a Central Apennine mountain (Mount Genzana): (**a**) Hemicryptophytes, (**b**) Chamaephytes, (**c**) Phanerophytes, (**d**) Geophytes, (**e**) Therophytes. Dashed lines are parabolic or monotonic fit obtained with generalized linear models. Fit parameters are reported in Table 2.

Proportion of hemicryptophytes increased monotonically with elevation (Figure 5a, Table 2). Proportion of chamaephytes varied with elevation with a clearly U-shaped pattern (Figure 5b, Table 2), whereas both phanerophytes (Figure 5c, Table 2) and geophytes (Figure 5d) showed opposite, hump-shaped patterns. Therophytes were not fitted because of their small number (only three species sampled) and lack of any pattern (Figure 5e).

**Figure 6:**
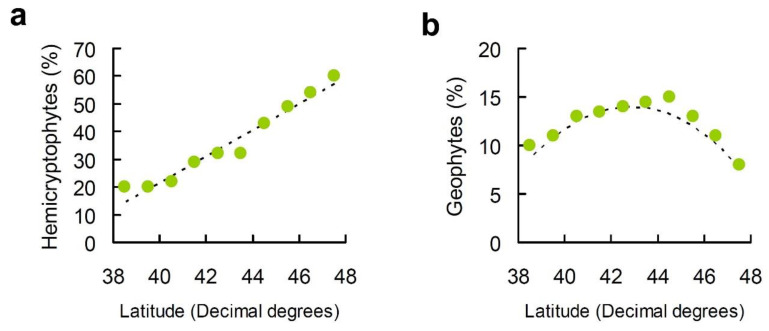
Relationship between percentage of (**a**) hemicryptophytes and (**b**) geophytes and latitude on a Central Apennine mountain (Mount Genzana) fitted using conditional autoregressive models.

On the Italian peninsula, the percentage of hemicryptophytes increased with latitude (Figure 6a; CAR model: −166.727 + 4.713 × Latitude, *R*^2^ = 0.954, *p* < 0.001), whereas geophytes showed a hump-shaped pattern (Figure 6b; CAR model: 499.632 + 23.986 × Latitude – 0.280 × Latitude^2^, *R*^2^ = 0.929, *p* < 0.001).

## 3. Discussion

The majority of investigations on species richness variation along elevational gradients found either a monotonic decline with increasing elevation or a hump-shaped pattern with a mid-elevational peak [15,21,65,66,67,68,69,70,71,72,73]. The monotonic decline is generally explained as a consequence of increasingly harsher conditions, lower productivity, less available area (due to the conical shape of the mountains) and the nested distribution of species, with species at higher elevations that tend to be subsamples of the more tolerant species already occurring at lower elevations [15,21,83,84,85,86,87,88]. The hump-shaped pattern is usually considered the result of the fact that species distribution tends to overlap at the domain centers due to dispersal restrictions (mid-domain effect) [15,21]. Some authors think that the hump-shaped pattern is probably the most widespread one [89,90], and that the monotonic decrease may be due to reduction in the available area with elevation [91].

In our study system, we found that species richness varied with elevation following a distinctly hump-shaped relationship. An analysis of the surface available at different elevations indicates that, quite surprisingly, it did not decline monotonically, but peaked at the middle of the gradient, with about 47% of surface concentrated between 1000 m and 1500 m. This can be explained by the very inner position of the study area within the Central Apennines, where low elevations are rare. This peculiar situation raises the possibility that the observed hump-shaped pattern might be due to the corresponding available surface at mid elevations. However, our results demonstrated that the hump-shaped pattern persisted even after correcting species richness for available area.

Our results contrast with the monotonic decrease in species richness reported for Apennine mountains by Theurillat et al. [33] for the flora of Mount Velino and by Di Musciano et al. [41] for the Apuan Alps. However, the gradient investigated by Theurillat et al. [33] spanned from 1100 m to 2400 m, thus corresponding to the section of our gradient showing a declining richness. In general, it is not rare to find linear patterns along relatively short gradients [92], whereas unimodal patterns are more likely to be found for the responses of species or functional groups when gradients are long [16,18]. On the other hand, the linear decrease found by Di Musciano et al. [41] may be explained by that fact that their study system was a large mountain chain facing the Tyrrhenian Sea, thus hosting many Mediterranean species linked to lowlands which likely decrease with elevation.

In accordance with our predictions, the analysis of the chorotypes in our study system revealed a prevalence of European and Euromontane species, whose relative richness varied along the elevational gradient with a hump-shaped pattern. The prevalence of these species in the study gradient, which ranges from 600 m to 2000 m, suggests that they are mainly mesophylous and montane species, which are especially advantaged by climatic conditions at middle elevations of the study area, and offers a biogeographical explanation for the observed hum-shaped pattern in the species richness.

Overall, the species more or less widely distributed in Eurasia (i.e., the European, Euromontane and Euro-Asiatic species) accounted for more than 50% of the sampled flora, while the Mediterranean species (i.e., Eurymediterranean, Mediterraneo-Montane, and Stenomediterranean species) were about 22%. Thus, despite the location of the study area in the middle of the Mediterranean basin, the biogeographical composition of its flora is influenced much more by Euro-Asiatic species than by Mediterranean species. This pattern, which is consistent with what can be observed on a regional scale [74], suggests that the flora of the study area was profoundly shaped by species that were favored by Pleistocene glaciations, which allowed many cold-adapted and cold-tolerant species of Europe and Asia to colonize the Italian peninsula, and which, after deglaciation, Pleistocene retreated into mountain areas [74]. The decline of European and the Euro-Asiatic species in the upper part of our gradient suggests that they are represented by species that benefit from a temperate climate, but which are unable to survive the severe cold conditions of the higher elevations. On Mount Velino, Theurillat et al. [33] found a decrease in the proportion of European and Euro-Asiatic species along the gradient, which extended from 1100 m to 2400 m. These patterns are therefore perfectly consistent with the decline observed for the European and Euro-Asiatic species from 1000 m to 2000 m in our more extended gradient.

As expected, we observed a decline of species with Mediterranean distributions along the gradient. The Stenomediterranean and the Eurymediterranean species dominated the species assemblages below 800 m, which is consistent with our predictions and suggests that the climatic conditions at lower elevations favor species able to cope with summer stressful conditions. A substantial decrease of Mediterranean species with elevation matches the pattern observed on Mount Velino [33], where Mediterranean species decreased regularly with increasing elevation. This decrease is also consistent with that found in the percent cover of Mediterranean species on a larger scale by Olthoff et al et al. [18] along a latitudinal-altitudinal gradient in Northern Spain. However, while the Eurymediterranean species had a rather homogeneous decline in their proportion with elevation, the Stenomediterranean species showed a very rapid decline followed by a very slight recovery at the higher elevations. This suggests that, after being rapidly decreased by increasing colder conditions, they are represented at high elevations by few, particularly resistant species which constitute a non negligeable fraction of the species-poor communities that live there.

The relatively high percentages of the Euromontane and Mediterraneo-Montane species found in our study is in accordance with the geomorphology of the study area, which has most of its surface (more than 77%) over 1000 m. As expected, Euromontane and Mediterraneo-Montane species distinctly increased with elevation, which is an obvious reflection of their montane character. However, while the Mediterraneo-Montane species increased with elevation over the entire gradient, the Euromontane were best fitted by a parabolic model, which is a reflection of the presence of orophytes distributed in South European and North Mediterranean areas. Theurillat et al. [33] included the Mediterraneo-Montane species among the Mediterranean ones, whereas we preferred to distinguish them because of their preference for montane habitats, which is reflected by their increasing proportion with elevation. In fact, from a biogeographical point of view, the Mediterraneo-Montane species (i.e., Mediterranean orophytes) are more related to the Euromontane species (i.e., South European orophytes) than the Mediterranean ones. The temperate and warm areas of South Europe are studded with more or less isolated massifs of Tertiary origin (such as the Sierra Nevada, the Pyrenees, the Alps, the Apennines, the Carpathians, the Balkans, etc.). Part of the Mediterranean flora colonized these montane areas, evolving as orophytes both in South European and Mediterranean chains [74].

Endemic species (which include some Apennine endemics) were a conspicuous fraction of the sampled flora (about 14%). This percentage is much higher than that recorded for the whole region of Abruzzi (about 8% based on Peruzzi et al. [93] and Bartolucci et al. [94]) but similar to that observed for the flora of the Central Apennines above the tree line (13% [75]). The high incidence of endemics in the sampled flora can be explained by the role of mountain regions as ecological archipelagos promoting evolutionary processes [6,21,67,95,96]. Endemic species increased their proportion with altitude, consistent with our predictions and the pattern observed by Theurillat et al. [33] on Mount Velino and by Di Musciano et al. [41] on the Apuan Alps, and with the hypothesis that endemics should be common at higher elevations [97,98]. In our study gradient, all endemics are species restricted to mountain areas of Italy, being mostly distributed over 1000 m (one, *Cynoglossum magellense*, occurs almost exclusively above 2000 m), and are mainly associated with prairies, being therefore possibly advantaged by a moderate grazing. Thus, the increasing proportion of endemics with elevation can be the reflection of both biogeographical and ecological reasons.

Species with wide distributions are typically indicative of areas with anthropogenic disturbance or occupied by azonal vegetation [99] or characterized by transitional environments [16,18]. The small proportion of species with wide distribution (Cosmopolitan, Boreal and Paleotemperate) in the study area is in line with the fact that, despite the presence of pastures, disturbance is relatively reduced there. However, whereas Cosmopolitan and Boreal species did not show any clear elevational pattern, the Paleotemperate species followed a hump-shaped pattern, which indicates that they have ecological preferences and biogeographical histories similar to those of the European and Euro-Asiatic species.

The proportions of life forms in the study showed a prevalence of hemicryptophytes and a very low presence of therophytes, which contrasts markedly with in the situation observed on the whole Italian territory where the therophytes are the most abundant group after the hemicryptophytes [74]. Thanks to their ability to cope with thermo-xeric climatic conditions, therophytes are the regionally richest group in Mediterranean areas [20,40]. In these areas, the therophytes are mainly associated with warm and dry lowland sites [20,40,100] with a high degree of precipitation seasonality [100], and decline sharply with elevation, which suggests a low ability to tolerate decreasing temperature and increasing precipitation [20,33,40,41,101,102]. The small number of therophytes recorded in our study system can be therefore explained by the mountainous character of the study area (as expected), and is in line with the small number of annual species typically recorded at high elevations, where they usually represent less than 2% of the total alpine flora [6,41]. Therophytes tend to prevail in disturbed habitats [40,103,104], increasing their proportion in heavily grazed areas [42,105]. Thus, the scarcity of therophytes in our samples is also consistent with the low incidence of anthropogenic pressures in the study area. By contrast, the relatively high proportion of therophytes on Mount Velino (about 17% [33]) may be explained by the strong impact of grazing in this area. It has been suggested that a ratio of hemicryptophytes to therophytes <1 is indicative of a Mediterranean character of the flora, while a ratio > 1 indicates a continental nature [106], with the vegetation in the temperate zones being dominated by hemicryptophytes [37]. In our case, the ratio was 49, which indicates a strongly continental character of the sampled flora.

The prevalence of hemicryptophytes and chamaephytes in our study system follows our predictions, and it is consistent with their dominant role commonly observed in high mountain ecosystems [6,33,38,40,42,77,107]. In general, it has been observed that the proportion of hemicryptophytes increases with elevation [33,36,40,41,108] in response to increasing precipitation and decreasing temperature [40,108], with this life form being the most dominant at high elevations [109,110]. In accordance with this general pattern, in our study area the hemicryptophytes increased monotonically with elevation (as expected), representing almost 80% of the species at the highest altitudes.

The position of buds close to soil surface may offer the hemicryptophytes some protection from the harsh climatic conditions of higher elevations [78], especially during winter when the perennial structures are protected by the snow. Low disturbance conditions at higher elevations may favor the hemicryptophytes [42], which are less tolerant of grazing than species with subterranean over-wintering buds [111,112].

Chamaephytes are known to be the predominant life form in cold and dry climates, where these plants overwinter thanks to the higher temperature of the soil and the protection of the snow that covers them during winter [6]. These characteristics make the chamaephytes able to complete their life cycle even in the short growing periods occurring at high elevations [113,114]. Chamaephytes are therefore able to successfully colonize high-altitude environments, and various research studies highlighted an increase in their proportion with increasing elevation [20,33,40,41]. However, a U-shaped pattern, with a minimum at the mid-mountain, has been also observed in some contexts [37,115], albeit reasons for this pattern remain elusive. The parabolic pattern observed in our study system might be explained with our hypothesis of the influence of the “double-stress” stress gradient that characterizes Mediterranean mountains. At high elevations, chamaephytes are advantaged by their ability to cope with low temperatures, while at low elevations they can be favored by their tolerance to drought (about 33% of the chamaephytes recorded in our sampled flora are Eurymediterranean and Stenomediterranean species), thus originating a U-shaped pattern. The positive relationship between percentage of chamaephytes and elevation found on Mount Velino [33] may be due to the absence of sampling sites below 1000 m.

Proportion of phanerophytes (about 12%) in our study was slightly higher than those recorded for the whole Italian territory [74], the Abruzzi-Molise region [74] and Mount Velino [33] (about 9% in all cases). In our study, the phanerophytes showed a distinct hump-shaped pattern, which is consistent with our predictions. Di Musciano et al. [41] did not find any relationship between the proportion of phanerophytes and elevation on the Apuane Alps, whereas Theurillat et al. [33] found a linear decrease. Since the gradient investigated by these latter authors starts from 1100 m, this decline corresponds with the declining part of our pattern. Notably, Lazarina et al. [20] found, on an altitudinal gradient ranging from 0 m to 1500 m on Crete, a slightly increasing pattern. A hump-shaped pattern was, however, found by Olthoff et al et al. [18] for percent cover along a latitudinal-altitudinal gradient in Northern Spain. In general, the percentage of woody plants tends to be positively influenced by increasing moisture conditions [116], and hence it tends to increase with elevation [40,117]. This explains the increase in their proportion from 500 m to about 1000 m observed in our study. The highest proportion of phanerophytes observed at mid elevations corresponds to the concentration of woodlands there. Thanks to their large structures, phanerophytes have a competitive advantage over other plant life forms under the humid and mild conditions of intermediate elevations but become poor competitors at higher elevations. In our study area, the tree line was at about 1800 m, and the only phanerophyte recorded above this altitude was the juniper *Juniperus sibirica*.

Geophytes represented a small fraction (less than 10%) of the sampled flora, in line with their proportion in the whole Italian flora (about 12%) and the Abruzzi and Molise regional flora (about 13%) [74]. As expected, geophytes showed a hump shape, peaking at around 1000 m, a pattern which is consistent with that observed in Italy by Theurillat et al. [33] on Mount Velino (where geophytes were 11%) and by Di Musciano et al. [41] on the Apuan Alps. In other areas, the relationship between geophytes and elevation followed either a monotonic increase [20,37] or a monotonic decrease [34,40,118]. Danin and Orshan [118] and Procheş et al. [119] found that geophytes increased along a precipitation gradient in Mediterranean ecosystems, suggesting that they are favored by a high degree of precipitation seasonality, but Irl et al. [40] found an opposite pattern. Geophytes are common in forest habitats [120] and in Italy they are mainly associated with deciduous mixed woodlands and beech forests where bulbous and rhizomatous species are frequent [74]. Thus, their prevalence at mid elevations in our study system may be explained by the occurrence of these forms of vegetation there. Specifically, our relevés with the higher proportions of geophytes were in mixed mesophylous and semimesophylous woods and in the beech forest [79].

Elevational patterns of hemicryptophytes (monotonic increase) and geophytes (unimodal) were paralleled by those observed along the latitudinal gradient in the Italian peninsula, thus supporting our expectations. Temperatures declined with both elevation and latitude, but changes were more rapid along the elevational gradient than the latitudinal one (the world average is a drop of about 1 °C for every 150 m above sea level against 1 °C for every 150 km poleward [6,21]). On the Italian peninsula, the percentage of hemicryptophytes increased from 20% to about 60% along a latitudinal gradient of 900 km. Assuming the aforementioned declines in temperature, this latitudinal gradient should translate to an elevational gradient of about 900 m. Very consistently, we found that in our elevational gradient the hemicryptophytes increased from about 25% (at 620 m) to about 75% (at 1630 m). The correspondence between elevational and latitudinal variation in vegetation is one of the earliest ecological patterns to have been discovered [3] and these findings support the possibility of extending such parallelism to life forms as hypothesized [81].

## 4. Materials and Methods

### 4.1. Study Area and Data Sources

We used published data [79] from a floristic/phytosociological study on the vegetation of “Monte Genzana e Alto Gizio” natural reserve (3160 hectares), which includes the Monte Genzana calcarean massif (41°56′53.37″ N–13°53′14.91″ E) and it is almost entirely included in the Special Area of Conservation “Monte Genzana IT7110100 ZSC” territory. The massif is located in the inner part of Central Apennines and is characterized by a hilly and mountainous landscape, with an elevation range spanning from 530 m to 2170 m on the summit of Monte Genzana. The whole area includes only a small, inhabited center (Pettorano sul Gizio), which, however, occupies a very peripheral portion of the reserve, and paved roads are virtually absent. Anthropic pressure is mainly represented by the presence of grazing zones with dedicated structures, such as sheepfolds and watering places, and by coppicing activities as a wood management strategy. Thus, despite the long history of human presence in the Apennines, the area is characterized by a high degree of wilderness. Most of the territory is covered by woods, followed by grazed or fallow lands.

Due to its inner position and montane characteristics, the area has a temperate-continental climate. According to the phytoclimatic map of Italy [121], most of the area is included in the classes Supratemperate/Mesotemperate hyperhumid/humid (temperate oceanic bioclime, hyperhumid hombrotype) and Supratemperate hyperhumid/ultrahyperhumid (temperate oceanic-semicontinental bioclime, hyperumid-oromediterranean hombrotype); a smaller fraction of the territory is included in the classes Supratemperate/Mesotemperate humid/hyperhumid (oceanic semicontinental bioclime, subhumid hombrotype) and Mesotemperate humid/subhumid (temperate oceanic-semicontinental bioclime, subhumid hombrotype).

Thanks to the remarkable extent of its elevational range, this area shows a representative cross-section of the Central Apennines encompassing forms of vegetation from all vegetational belts that can be found on the Apennines: thermophilic woods in the lowlands and hilly lands, dominated by downy oak (*Quercus pubescens*) and European hop-hornbeam (*Ostrya carpinifolia*); beech forests (from 1000 to 1800 m); subalpine shrublands; and high-montane grasslands.

We used data on species distribution from 16 relevés [79] along the whole elevational gradient within the study area (from about 600 m to 2000 m). Taxonomy was revised and updated following Pignatti et al. [122] (see Table S1).

All plant species recorded were initially assigned to their respective chorotype as coded by Pignatti et al. [122]. However, to provide detailed description of species ranges, Pignatti et al. [122] used a very large number of chorotypes, many of which describe very similar patterns. Thus, we grouped these original chorotypes into ten major groups [33,74,123] (hereafter, the word “chorotype” will refer to these groups): (1) Boreal, (2) Cosmopolitan, (3) Euro-Asiatic, (4) European, (5) Eurymediterranean, (6) Stenomediterranean, (7) Mediterraneo-Montane, (8) Euromontane, (9) Paleotemperate and (10) Endemic (Table S1).

Each species was assigned to a Raunkiaer’s life form (chamaephytes, geophytes, hemicryptophytes, phanerophytes, and therophytes) using Pignatti et al. [122]. Nanophanerophytes (i.e., phanerophytes smaller than 2 m) were included among the phanerophytes. When for a given species Pignatti et al. [122] listed more than one life form, we retained the more durable one (for example, between phanerophyte and chamaephyte we retained the former, whereas between therophyte and hemicryptophyte we retained the latter) [41] (Table S1). However, this occurred only for a minority of the species (16 out of 250, less than 7%).

Frequencies of life forms in Italian regions were taken from Pignatti [74]. To model the latitudinal variation in the relative frequencies of life forms we used maps provided by Pignatti [74] for hemicryptophytes and geophytes, divided the Italian peninsula into latitudinal bands of one degree, and calculated average percentages per band (Pignatti [74] did not provide maps for chamaephytes and diversity of therophytes was very low in our study area).

### 4.2. Study Area and Data Sources

The species–elevation relationship was modelled with a parabolic fit using an ordinary least squares (OLS) regression. Since the species richness recorded at the scale of the relevés can be influenced by the overall number of species occurring at the elevation from which local sampling was conducted, and this overall richness can be, in turn, influenced by the area available at that elevation [72], we also used here the approach proposed by McCain [124] to control for variation in area availability at different elevations. For this, we divided the study area into 50 m elevational belts and used a digital elevational model to calculate the amount of land surface in each belt. Then we assigned to each relevé the area of the corresponding elevational band. Finally, assuming a power function *S = cA^z^* for the species area relationship (where *S* is the number of species and *A* is area), we calculated the constant *c* (*c* = *S/A^z^*), which expresses the number of species per area unit, and regressed *c*-values against elevation. For the *z*-value we used the canonical value of Preston (0.25) [124,125].

To investigate how the proportion of chorotypes and life forms varied along the gradient, we used generalized linear models (GLMs) with a binomial distribution of errors (or a quasibinomial distribution in case of overdispersion). Models were implemented as unimodal relationships (*y ~ x + x*^2^) and the quadratic term dropped based on Akaike information criterion (AIC) [40]. For fitting purposes, we used raw polynomials, whereas orthogonal polynomials were used for calculation of *p*-levels. To model variations in the percentages of life forms with latitude along the Italian peninsula, conditional autoregressive (CAR) models were used. OLSs and GLMs were performed using R version 3.5.2. [126] CAR models were performed using SAM 4.0 [127].

## 5. Conclusions

We found a general hump-shaped pattern of plant species richness along the elevational gradient, which can be explained by the harsher conditions that characterize the lowest and the highest elevations. This pattern is distinctly related to prevalence at mid elevations of species with European and Euro-Asiatic distributions, which are favored by temperate climatic conditions. As mid elevations are largely occupied by woodland vegetation, this explains the prevalence in this part of the gradient of phanerophytes and geophytes (which are mainly associated with woods). The observed increase in the hemicryptophytes with elevation is explained by their ability to cope with high elevation climatic conditions. Species with Mediterranean distributions prevail at the lowest elevation but decline with elevation, because they are negatively affected by decreasing temperatures. Chamaephytes showed a U-shaped pattern, suggesting they are able to cope with arid and cold conditions at the extremes of the gradient. Finally, the endemics increased with elevation, as expected on the basis of their association with mountainous areas as key places for endemism evolution. These results illustrate how elevational patterns in species richness, biogeographical composition and life forms are interrelated and provide reciprocal insights for understanding current vegetation settings.

## Figures and Tables

**Table 1:** Summary statistics of fitted polynomial generalized linear models for proportion of plant chorotypes along an elevational gradient on a Central Apennine mountain (Mount Genzana). Error refers to Standard Errors. Model goodness-of-fit is expressed as McFadden’s psedudo-*R*^2^. * = *p* < 0.05; ** = *p* < 0.01; *** *p* < 0.001.

Chorotype	Intercept	Elevation	Elevation^2^	Pseudo-*R*^2^
European	−4.38 ± 1.23 ***	6.09 × 10^−3^ ± 2.06 × 10^−3^ *	−2.49 × 10^−6^ ± 7.73 × 10^−7^ ***	0.56
Euro-Asiatic	−10.23 ± 2.32 ***	1.37 × 10^−2^ ± 3.66 × 10^−3^ *	−5.21 × 10^−6^ ± 1.33 × 10^−6^ ***	0.51
Paleotemperate	−4.91 ± 2.09 ***	4.41 × 10^−3^ ± 3.51 × 10^−3^	−1.86 × 10^−6^ ± 1.32 × 10^−6^	0.38
Mediterraneo-Montane	−3.77 × ± 0.64 ***	8.57 × 10^−4^ ± 3.86 × 10^−4 ^*	-	0.25
Endemic	−4.17 ± 0.61***	1.44 × 10^−3^ ± 3.5 × 10^−4^ ***	-	0.55
Euromontane	7.38 × 10^−2^ ± 1.48 ***	−4.83 × 10^−3^ ± 2.51 × 10^−3^ ***	2.22 × 10^−6^ ± 9.32 × 10^−7^ **	0.71
Stenomediterranean	8.84 ± 2.36 ***	−1.97 × 10^−2^ ± 4.66 × 10^−3^ ***	6.91 × 10^−6^ ± 1.81 × 10^−6^ ***	0.81
Eurymediterranean	−0.53 ± 0.37	1.09 × 10^−3^ ± 3.00 × 10^−4^ ***	-	0.57

**Table 2:** Summary statistics of fitted polynomial generalized linear models for proportion of plant chorotypes along an elevational gradient on a Central Apennine mountain (Mount Genzana). Error refers to Standard Errors. Model goodness-of-fit is expressed as McFadden’s psedudo-R2. * = *p* < 0.05; *** *p* < 0.0001.

Life form	Intercept	Elevation	Elevation^2^	Pseudo-*R*^2^
Hemicryptophytes	−1.36 ± 0.40 ***	1.26 × 10^−3^ ± 2.67 × 10^−4^ ***	-	0.62
Chamaephytes	7.96 ± 1.54 ***	−1.63 × 10^−2^ ± 2.82 × 10^−3^ *	6.07 × 10^−6^ ± 1.07 × 10^−6^ ***	0.72
Phanerophytes	−10.50 ± 2.75 ***	1.85 × 10^−2^ ± 5.11 × 10^−3^ ***	−8.79 × 10^−6^ ± 2.32 × 10^−6 ^***	0.80
Geophytes	−14.30 ± 3.12 ***	2.02 × 10^−2^ ± 4.83 × 10^−3^	−7.74 × 10^−6^ ± 1.75 × 10^−6^ ***	0.73

## Data Availability

All data used in this study are provided as Supplementary Materials (Table S1).

## Supplementary Materials

The following are available online at https://www.mdpi.com/article/10.3390/plants10102090/s1, Table S1. Species distribution, chorotypes and life forms.
